# Liver Resection Under Ischemia: Inflow Occlusion or Total Hepatic Isolation

**DOI:** 10.1155/1995/19753

**Published:** 1995

**Authors:** Bengt Jeppsson

**Affiliations:** Department ofSurgery Lund University Hospital Lund S-221 85 Sweden


surgery group. The procedure-related complications (28% and 32%, respectively) and 30 day mortality (8% and 20%) were similar. Although the initial hospital stay was significantly shorter in the stented group (18 vs 24 days), this difference was not maintained when readmissions for obstructed endoprostheses and duodenal obstruction were also considered. Another study by Dowsett *et al.* included 127 patients with unresectable malignancy obstructing the *distal* bile duct[12]. Sixty-five patients were treated via endoscopic stenting and 62 had surgical palliation. Successful biliary drainage was achieved in 94% of patients, with the 30 day mortality being 6% after endoscopy and 15% after surgery. However, recurrent jaundice (17% vs 3%) and late duodenal obstruction (14% vs 3%) were seen more commonly in the endoprosthesis group. Unfortunately, no prospective randomized studies have compared surgical to nonsurgical palliation of *hilar* cholangiocarcinoma or more proximal biliary obstruction, and therefore data applicable to this situation are lacking.

In conclusion, the management of patients with obstructive jaundice from either benign or malignant processes no longer resides solely within the hands of surgeons. A multidisciplinary approach to such a problems is currently indicated, with some patients being best treated by endoscopic or percutaneous techniques, others by surgical techniques, and still others using a multidisciplinary approach. Operatively placed transhepatic stents continue to play an important role in the management of these patients. So do the procedures performed by our talented endoscopists and interventional radiologists.

*References*

1. Terblanche, J., Kahn, D., Bornman, P. C., Werner, D. (1988) The role of U tube palliative treatment in high bile duct carcinoma. *Surgery*, **103**, 624–632.
2. Cameron, J. L., Broe, P., Zuidema, G. D. (1982) Proximal bile duct tumors. Surgical management with silastic transhepatic biliary stents. *Ann. Surg.*, **196**, 412–419.
3. Yeo, C. J. and Cameron, J. L. (1992) Transhepatic biliary stents in high benign and malignant biliary tract obstructions. In: *Mastery of Surgery*, 2nd edition, Nyhus, L. M. and Baker, R. J. (ed.), Boston, Little Brown and Co., 960–967.
4. Davidoff, A. M., Pappas, T. N., Murray, E. A. *et al.* (1992) Mechanisms of major biliary injury during laparoscopic cholecystectomy. *Ann. Surg.*, **215**, 196–208.
5. Rossi, R. L., Schirmer, W. J., Braasch, J. W. *et al.* (1992) Laparoscopic bile duct injuries. Risk factors, recognition and repair. *Arch. Surg.*, **127**, 596–602.
6. Ress, A. M., Sarr, M. G., Nagorney, D. M. *et al.* (1993) Spectrum and management of major complications of laparoscopic cholecystectomy. *Am. J. Surg.*, **165**, 655–662.
7. Lai, E. C. S., Chu, K. M., Lo, C. Y. *et al.* (1992) Surgery for malignant obstructive jaundice: Analysis of martality. *Surgery*, **112**, 891–896.
8. Ottow, R. T., August, D. A., Sugarbaker, P. H. (1985) Treatment of proximal biliary tract carcinoma: An overview of techniques and results. *Surgery*, **97**, 251–262.
9. Cameron, J. L., Pitt, H. A., Zinner, M. J. *et al.* (1990) Management of proximal cholangiocarcinomas by surgical resection and radiotherapy. *Am. J. Surg.*, **159**, 91–98.
10. Davids, P. H. P., Groen, A. K., Rauws, E. A. J. *et al.* (1992) Randomized trial of self-expanding metal stents versus polyethylene stents for distal malignant biliary obstruction. *Lancet*, **340**, 1488–1492.
11. Bornman, P. C., Harries-Jones, E. P., Tobias, R. *et al.* (1986) Prospective controlled trial of transhepatic biliary endoprosthesis versus bypass surgery for incurable carcinoma of the head of the pancreas. *Lancet*, **1**, 69–71.
12. Dowsett, J. F., Russell, R. C. G., Hatfield, A. R. W. *et al.* (1989) Malignant obstructive jaundice: A prospective randomized trial of bypass surgery versus endoscopic stenting. *Gastroenterology*, **96**, A128.

J. Charles Yeo, M.D.
Department of Surgery; Blalock 606
The Johns Hopkins Hospital
600 N. Wolfe Street
Baltimore, MD 21287-4606
Phone No: (410) 955-7496
FAX No: (410) 614-3539

### LIVER RESECTION UNDER ISCHEMIA: INFLOW OCCLUSION OR TOTAL HEPATIC ISOLATION

### ABSTRACT

*Huguet, C., Gavelli, A., Chieco, P. A., Bona, S., Harb, J., Joseph, J. M., Gramaglia, M. and Lasserre, M. (1992) Liver ischemia for hepatic resection: Where is the limit? Surgery; III: 251–259.*

***Background.*** **A consecutive series of 50 patients who submitted to 53 hepatic resections with use of continuous normothermic liver ischemia is reported.**
***Methods.*** **Portal triad clamping has been used in 28 cases, with associated inferior vena caval clamping above and below the liver (hepatic vascular exclusion) in 25 patients. The size of the tumor required major hepatic resection in 38 cases (71.7%). Malignant tumors (83%) were the most common indication for liver resection. Patients were placed in three groups according to the duration of liver ischemia: group A, less than 30 minutes (9 patients); group B, 30 to 60 minutes (29 patients); and group C, 60 or more (15 patients).**
***Results.*** **No difference in mortality rates (5.7% in the entire series and 0% in group C) and morbidity rate could be shown. No significant difference was found in postoperative liver test results, and no persistent alteration remained thereafter. Liver biopsy at 6 and 12 months after operation did not reveal any chronic damage. Liver capability to regenerate was maintained as documented by postoperative computerized tomography scan or magnetic resonance imaging.**
***Conclusions.*** **Because interruption of hepatic blood flow in normothermia is safe for atleast 60 minutes (up to 85 minutes in this study), vascular clamping is recommended for hazardous liver resections to minimize blood loss, which appears to be the main factor of death and morbidity. (SURGERY 1992; 111: 251–259.)**

KEY WORDS: Liver ischemia hepatic resection.

### PAPER DISCUSSION

This is a well-written report on portal triad clamping alone or with associated inferior vena caval clamping (hepatic vascular exclusion). It is from a centre with a vast experience of this type of surgery and therefore the results are extremely interesting. The main indication for using vascular exclusion is to reduce blood loss and the authors give some data to support this. During transection of hepatic parenchyma haemostat fracture technique was used. The reduction of blood loss has been challenged later by a group from Japan[1]. In this study liver resection was performed with and without vascular occlusion and the authors could not detect any significant differences between the groups in blood loss or blood transfusion requirement. The authors conclude that the reason for this is a safe dissection of hepatic parenchyma using an ultrasonic surgical aspirator and electric coagulator. So the issue of reduced blood loss is still not settled but up to now most authors would agree that vascular occlusion will reduce blood loss at least during surgery.

The authors ask the question: where is the limit? and that is the main object of the report. After reading the article, it is obvious that the limit definitely goes beyond 60 minutes for tolerance of the liver for normothermic ischemia. The 30 days mortality was 1.9% and the in-hospital mortality rate was 5.7%. These are figures that compare favourably with results from other specialized centres. The conclusion from the paper is therefore that normothermic ischemia for at least one hour can be recommended in liver surgery to decrease the bleeding complication. However, the major complication rate was 22.6% and the minor complication rate was 30.2%. In another paper with a similar approach a lower mortality rate was also observed but complications were observed in almost 50% of the patients[2].

The issues concerning prolonged normothermic ische-mia of the liver are twofold: first the tolerance of the hepatocytes and Kupffer cells to ischemia and second the injurious effect on the small and large bowel mucosa of occlusion of the portal vein. Apparently the liver, even if it is cirrhotic can withstand pure ischemia for a period up to 90 minutes without significant cellular injury[3]. The harmful effect of portal occlusion and intestinal stasis has, however been less clearly defined. The complication rate and morbidity rate in liver transplantation and after elective hepatic surgery are about 30% in most published series[4]. Most complications are of septic origin and enteric bacteria seem to be involved. There is an increasing body of evidence indicating that portal stasis will induce an increased translocation of gut bacteria to the portal circulation[5] and there is an indication of increased bacterial translocation from the gut during liver resection[6]. This may then occur in a situation where Kupffer cells are already under stressful influence by ischemia. These two events may combine to induce the increased morbidity rate. Therefore a word of caution must be expressed when interpreting the result of normothermic ischemia of the liver during liver resection and less favorable results may be obtained if this technique gives wide-spread acceptance at centres with less experience of liver surgery. For the more research should be directed at studies of the interplay between bowel integrity, portal circulation and liver function. Different preventative measures to increase the gut integrity in the form of selective antibiotic treatment, administration, of immunostimulants, stimulation of mucosal growth or induction of bacterial antagonism by the administration of probiotics require further evaluation.

With this in mind the answer to the question posed in the paper could be within the range of 60-90 minutes but the question should be extended: where is the limit for combined liver ischemia and splanchnic stasis?

*References*

1. Taniguchi, H., Takahashi, T., Shioaki, Y., Itoh, A., Oguro, A. (1992) Vascular inflow exclusion and hepatic resection. *Br. J. Surg.*, **79**, 672–675.
2. Hannoun, L., Borie, D., Delva, E., Jones, D., Vaillant, J-C., Nordlinger, B., Parc, R. (1993) Liver resection with normothermic ischemia exceeding 1 h. *Br. J. Surg.*, **80**, 1161–1165.
3. Kim, Y. I., Nakashima, K., Tada, I., Kawano, K., Kobayashi, M. (1993) Prolonged normothermic ischemia of human cirrotic liver during hepatectomy: a preliminary report. *Br. J. Surg.*, **80** 1566–1570.
4. Ekberg, H., Tranberg, K-G., Andersson, R., Jeppsson, B., Bengmark. S. (1986) Major liver resection-perioperative course and management. *Surgery*, **100**, 1–8.
5. Liu, L., Jeppsson, B., Bengmark, S. (1992) Bacterial translocation into portal circulation from the gut during portal triad occlusion. *Digestion*, **9**, 95–101.
6. Wang, X. D., Andersson, R., Soltesz, V., Bengmark, S. (1992) Bacterial translocation after major hepatectomy in patients and rats. *Arch. Surg.*, **127**, 1101–1106.

Bengt Jeppsson, MD, PhD
Associate Professor of Surgery
Department of Surgery
Lund University Hospital
S-221 85 LUND
SWEDEN
FAX: 46-46-13 97 76

### IS A CONSERVATIVE APPROACH JUSTIFIED IN PENETRATING LIVER INJURY?

### ABSTRACT

*Kundson, M.M., Lim, R.C. and Olcott, E.W. (1994) Morbidity and mortality following major penetrating liver injuries. Arch Surg; 129, 256–261.*

***Objective.*** **To establish the mortality and morbidity associated with major penetrating liver injuries and to describe the nature and treatment of complications related to these injuries. We postulated that there had been a trend toward less radical initial surgery, as well as an increased utilization of modern imaging techniques in both diagnosing and treating postoperative complications following penetrating liver trauma.**

***Design.*** **A retrospective survey of medical records and radiology files.**

***Setting.*** **A university trauma center in an urban setting.**

***Patients.*** **Of the 188 patients admitted to our trauma center with penetrating liver trauma between April 1988 and December 1991, 36 had major liver trauma (grades 3 through 5) and are described in this report.**

***Main Outcome Measures.*** **The mortality rate, type of operative treatment, and the nature and treatment of complications for each grade of major liver injury.**

***Results.*** **The mortality rate from major liver injuries was 17%. Surgical techniques employed primarily consisted of the use of hemostatic agents and cautery, simple suturing, direct vessel ligation, and packing. Fifty-two percent of the survivors had major complications related to the liver injury itself, but only two required operative therapy. The remaining patients were successfully treated with interventional radiologic techniques.**

